# Can Contraries Prompt Intuition in Insight Problem Solving?

**DOI:** 10.3389/fpsyg.2016.01962

**Published:** 2016-12-26

**Authors:** Erika Branchini, Ivana Bianchi, Roberto Burro, Elena Capitani, Ugo Savardi

**Affiliations:** ^1^Department of Human Sciences, University of VeronaVerona, Italy; ^2^Department of Humanities (Section Philosophy and Human Sciences), University of MacerataMacerata, Italy; ^3^Department of Education, Cultural Heritage and Tourism, University of MacerataMacerata, Italy

**Keywords:** insight, problem solving, contrast class, heuristics, contraries, spatial properties

## Abstract

This paper aims to test whether the use of contraries can facilitate spatial problem solving. Specifically, we examined whether a training session which included explicit guidance on thinking in contraries would improve problem solving abilities. In our study, the participants in the experimental condition were exposed to a brief training session before being presented with seven visuo-spatial problems to solve. During training it was suggested that it would help them to find the solution to the problems if they systematically transformed the spatial features of each problem into their contraries. Their performance was compared to that of a control group (who had no training). Two participation conditions were considered: small groups and individuals. Higher success rates were found in the groups exposed to training as compared to the individuals (in both the training and no training conditions), even though the time required to find a solution was longer. In general, participants made more attempts (i.e., drawings) when participating in groups than individually. The number of drawings done while the participants were trying to solve the problems did not increase after training. In order to explore if the quality (if not the number) of drawings was modified, we sampled one problem out of the seven we had used in the experiment (the “pigs in a pen” problem) and examined the drawings in detail. Differences between the training and no training conditions emerged in terms of properties focused on and transformed in the drawings. Based on these results, in the final discussion possible explanations are suggested as to why training had positive effects specifically in the group condition.

## Introduction

In this paper we explore the impact of explicitly guiding people to think in contraries when searching for solutions to visuo-spatial insight problems (examples of this kind of problem are provided in Table 1). We held a training session during which participants in the experiment were provided with demonstrations of how manipulating the representation of a problem in terms of contraries might be helpful. They were then asked to apply this “way of thinking” in a test phase where they were presented with other visuo-spatial problems. The aim of the experiment was to investigate the effects of specific hints or training on insight problem solving. The impact of general meta-cognitive training on performance has been addressed in previous literature (e.g., Walinga et al., 2011; Patrick and Ahmed, 2014; Patrick et al., 2015), as has the impact of more specific hints which have been customized to the contents of a specific problem (e.g., Chronicle et al., 2001, Experiment 3; Weisberg and Alba, 1981; Grant and Spivey, 2003; Kershaw and Ohlsson, 2004; Kershaw et al., 2013; Öllinger et al., 2013, 2014). What we aimed to focus on here, and to further test based on the results of the experiment, was the hypothesis that thinking in contraries might support transformations in the mental representation of a problem, as required by insight problem solving. Clear evidence of this has yet to be provided, but there are some precursors to the present study which suggest that the question is worth investigating. We will briefly revise these in the next section, contextualizing the underlying processing in terms of special-process and business-as-usual perspectives.

**Table 1:** **The seven problems used in the study**.

**Formulation**	**Figure**
**Pigs in a pen** (Schooler et al., 1993; Ball et al., 2015): participants are asked to construct two more square pens so as to ensure that each pig ends up in a pen of its own.	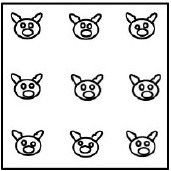
**Triangle** (De Bono, 1969; Schooler et al., 1993): participants are asked to make the triangle shape point downwards by moving only three circles.	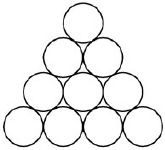
**Deer** (Origin unknown): participants are asked to make the deer face in a different direction by moving just one of the lines.	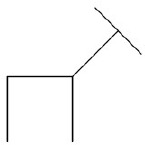
**Eight-coin** (Ormerod et al., 2002; Öllinger et al., 2013): participants are asked to move two coins in such a way that each coin only touches three other coins.	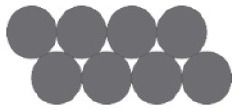
**Five-square** (Katona, 1940): participants are asked to reduce the number of squares from five to four by moving only three sticks.	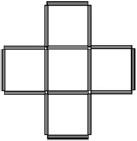
**Square** (Kanizsa, 1973): participants are asked to build a square by putting together six smaller figures: four right - angled isosceles triangles and two right - angled trapezoids of equal height but with bases of different lengths.	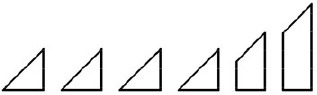
**Circumference** (Köhler, 1969): participants are asked to find the length of the oblique side CD, given the length of the diameter AB.	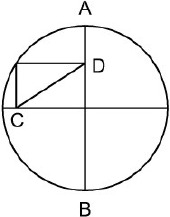

### Contrariety: a radical change while maintaining continuity

In the special process theory, insight is conceived of as a process arising from a sudden restructuring of the representation of a problem occurring at an unconscious level (Siegler, 2000; Kershaw and Ohlsson, 2004; Bowden et al., 2005; Murray and Byrne, 2013). From this point of view, insight is a discontinuous process since it implies a break with previous constraints and attempts. On the other hand, in the business-as-usual theory, insight is seen as a continuous, step by step, conscious process which is similar in nature to the processes underlying the solving of non-insight problems (Newell and Simon, 1972; MacGregor et al., 2001; Chronicle et al., 2004; Ormerod et al., 2013). An integrated perspective has also been put forward based on the argument that the two alternative views are not mutually exclusive and that they both contribute to insight although perhaps in different ways and/or at different moments (Fleck and Weisberg, 2013; Weisberg, 2015).

Using contraries as a strategy in problem solving seems to necessitate an integrated process of this type. Breaking things up into perceptual chunks and reorganizing them into opposite patterns means producing a radical change (i.e., a sharp discontinuity). However, at the same time this change is data-driven, that is, it is anchored on and driven by the inherent features of whatever is represented and in this sense the process implied is gradual. For instance, in Gale and Ball's studies (2003, 2006, 2009, 2012) on people's thought processes during hypothesis testing in Wason's (1960) 2-4-6 rule discovery task, the participants' performance improved when they were given a contrast class cue. In the original form of the task associated with Wason's rule, participants are asked to discover the rule (known only to the experimenter) that in this case governs the production of series of three numbers. The rule is “any ascending sequence.” Participants are then given 2-4-6 as a seed triple and are asked to generate further series of three number, which are then assessed by the experimenter as either conforming or not conforming to the target rule. When the participants are confident, they announce that they have discovered the rule. In their studies, Gale and Ball (2003, 2006, 2009, 2012) used a dual-goal variant of this task, in which participants are asked to discover two complementary rules, one labeled “DAX” (i.e., the standard “ascending numbers” rule) and the other labeled “MED” (i.e., “any other triples”). The aim was to test whether providing “contrast class cues” for the MED rule might facilitate participants' performance. They provided the participants with different types of contrast class cues as MED exemplars (see in particular Gale and Ball, 2012). One of these was the 6-4-2 triple, that contrasted with the original 2-4-6 triple on a salient and crucial dimension, i.e. an “ascending” series versus a “descending” series. The other exemplar series, i.e., 4-4-4 and 9-8-1, contrasted with the original series of numbers in terms of dimensions which were irrelevant to the task. Namely, 4-4-4 contrasts with 2-4-6 on the “same-different” dimension (i.e., “three identical numbers” versus “three different numbers”) while 9-8-1 contrasts with 2-4-6 on the “mixed-homogeneous” dimension (i.e., “mixed odd and even numbers” vs. “only even numbers”), as well as on the dimensions relating to “equal-unequal” intervals and whether the middle number “is-is not” the arithmetic mean of the two numbers which flank it. Participants who had been presented with examples of series of three ascending versus descending numbers recognized the oppositional nature of the two rules implied, explored fewer confirmatory alternatives and more frequently found the solution suggesting that contrasts play a facilitatory role. As a result of the evident contrast between the ascending and descending series of numbers, the thought process that was then triggered apparently focused on a marked *discontinuity*. However, at the same time, the cue also prompted the recognition of a straightforward relationship connecting the two example series suggesting that a *continuous* thought process was involved here too. In addition, a continuous, step by step process was suggested by the participants' tendency to generate from time to time hypotheses that varied along just one dimension. This latter feature is in agreement with hypothesis testing in general, as conceived by Oaksford and Charter in their iterative counterfactual model (1994).

The fact that there is a clear and straightforward relationship linking two “contrast classes” is part of the definition of this psychological construct (see how “contrast class” is defined by Oaksford and Stenning, 1992; Oaksford, 2002). More in general, the characterization of contrast/contrariety/opposition in terms of maximum distance with at the same time a high degree of affinity is a common feature in research in the fields of both Psycholinguistics and Experimental Psychology. In the areas of Cognitive Semantics and Linguistics, opposites refer to the *extremes* of an *underlying dimension* (e.g., Lehrer and Lehrer, 1982; Cruse, 1986; Jones et al., 2012). Antonyms are at the same time minimally and maximally different from one another. They are associated with the same conceptual domain, but they denote opposite poles or parts of that domain (Cruse, 1986; Paradis, 1997, 2001; Murphy, 2003, pp. 43–45; Willners, 2001; Croft and Cruse, 2004, pp. 164–192; Paradis et al., 2009). These two features (maximum distance and invariance) also characterize contrariety/opposition from a perceptual point of view. Various studies on the perception of this relationship in a number of different types of visual configuration have shown that a necessary condition for two events under observation to be perceived as contrary is that a maximum transformation of a salient feature (which in these studies was usually orientation) is manifested among overall invariant configurations. This has been formalized in the perceptual principles of non additivity and invariance in Bianchi and Savardi (2006; 2008a; see also Bianchi and Savardi, 2008b,c; Savardi et al., 2010; Bianchi et al., 2014).

The duality that thinking in terms of contraries seems to imply (i.e., maximum variation in an overall invariant configuration, extremes of a common underlying dimension and discontinuity in a clearly continuous pattern) also emerges when we consider negation and counterfactual thinking. In natural language and reasoning, humans tend to use negation in precise ways, following cognitive rules. One of the roles of negation is that of being a modifier of degree. This happens, for instance, when we say that “the water is not hot” about water that may be warm, lukewarm or cool (Bolinger, 1972; Horn and Kato, 2000; Israel, 2001; Giora et al., 2005a,b). Negation presupposes a polar dimension along which a shift away from the adjective to which not is applied occurs (Kaup et al., 2006, 2007; Paradis and Willners, 2006; Fraenkel and Schul, 2008; Bianchi et al., 2011c). Negated propositions are assumed to evoke two contrasting spaces, a factual and a counterfactual space (Langacker, 1991; Fauconnier and Turner, 2002; Hasson and Glucksberg, 2006). Counterfactual thinking requires a capacity to imagine alternatives to events, actions or states in order to test and validate hypotheses (Roese, 1997; Byrne, 2005). Counterfactual strategies are employed in falsification processes which are central to inductive and deductive reasoning (Wason, 1960, 1966, 1968; Farris and Revlin, 1989; Oaksford and Chater, 1994; Evans, 2002; Augustinova et al., 2005; Augustinova, 2008). In this case both confirmatory hypotheses and disconfirmatory hypotheses (that according to Wason's definition, 1960, literally *contradict* the previous ones) are generated. Counterfactual thinking is also implied in decision making. Inducing participants to take into account possibilities which are *diametrically opposite to their initial assumptions* is a de-biasing strategy which allows them to contrast the tendency to not consider adequately those alternatives which are at odds with their beliefs and perceptions and this leads to more accurate decisions (Lord et al., 1984; Mussweiler et al., 2000). The ability to *imagine contrasting alternatives* is also related to creativity and analytical problem solving. Additive counterfactuals, i.e. the addition of different antecedent elements to reconstruct reality (Roese and Olson, 1993), enhance performance in creative generation tasks that are facilitated by an expansive processing style, whereas subtractive counterfactuals, i.e., removing antecedent elements to reconstruct reality (Roese and Olson, 1993), enhance performance in analytical problem solving tasks that are facilitated by a relational process style (Markman et al., 2007).

The idea that both discontinuity and continuity are involved in the re-organization which takes place in insight problem solving was somehow prefigured by Gestalt psychologists. They did not explicitly discuss it in terms of contrariety/contrast, but in a sense they paved the way toward the hypothesis put forward in this paper, i.e., that contraries support the representational change that is required for an insight problem to be solved. As Wertheimer (1945) was the first to point out, a solution process requires problem solvers *to reorganize* the phenomenological features of the problem and this apparently occurs as a sudden “aha” moment. But it is less often remembered that Wertheimer also explicitly specified that this reorganization is based on the requirements of the initial phenomenological structure of the problem and is as such guided by them (representing the continuity element). According to him, the key operations in this reorganization are dividing elements that are unified and unifying elements that are separated while transforming their orientation and position in space [see Wertheimer's classic parallelogram problem, reported in Appendix 1 (Supplementary Material)]. From Duncker's perspective too (Duncker, 1945), productive thinking implies creating a break with the original formulation and representation of the problem and the usual way of thinking and using its inherent features in an unusual, sometimes even contrary way (representing the discontinuity element). In line with Wertheimer, he also explicitly stated that the solution process is suggested by and guided along directions emerging from the original structure of the problem.

If one adds to Wertheimer's and Duncker's premises the evidence that the human direct experience of space is grounded on oppositional structures which mostly refer to the human body (e.g., Howard and Templeton, 1966; Golledge, 1992; Shelton and McNamara, 1997; Tversky and Hard, 2009) such as near-far, high-low, vertical-horizontal, in front of-behind, above-below, left-right, etc. (Savardi and Bianchi, 2009; Bianchi et al., 2011a,b, 2013), one can see first of all why contraries support the transformation of a problem's spatial representation and foster its reorganization and secondly why they do so while remaining anchored to the structure of the problem (this has been partially discussed in Branchini et al., 2009, 2015b).

### Aware vs. unaware processes

One of the factors implied in business-as-usual versus special-process perspectives concerns the consciousness vs. unconsciousness of the underlying thought processes in problem solving. This is one of the basic dichotomies characterizing thinking and reasoning processes even beyond problem solving, as acknowledged in dual-process theories (for an updated review of this see Evans and Stanovich, 2013; Weisberg, 2015).

The issue is also discussed in the literature investigating the effects of hints or training in problem solving. In most of these studies the *hints* provided by the experimenters which aim to bring to the fore the critical feature of the problem consist of *implicit* suggestions to problem solvers. For example, the solution to Duncker's radiation problem speaks of multiple low-intensity lasers being directed from several angles tissue rather than concentrating them onto a limited area (and thus risking damage to the skin in that area). In Grant and Spivey's study (2003) the hint came from an animation of the whole oval perimeter representing the skin. In Bröderbauer et al.'s study (2013) on Katona's five square problem, the hint provided to participants in the experiment consisted of a “wave form” (the shape represented in the solution) hidden in the logo of the research group (Bröderbauer et al., 2013). In Öllinger et al.'s study (2013) on the eight-coin problem, the implicit suggestion to use the third dimension to find the solution was provided by a variety of different initial configurations of the eight-coin problem (some of which cued the use of the third dimension).

Conversely, *training* tends to work on an explicit level because the aim is to make participants aware of how to solve a specific set of problems. For example, Dow and Mayer (2004) developed four different training programs (i.e., a verbal insight problem training packet, a mathematical insight problem packet, a spatial insight problem training packet and a combined verbal and spatial insight problem training packet). Each of these included information about the critical features of the specific set of problems and a description of the three-step procedure to be followed in order to solve that particular type of problem. Patrick and Ahmed (2014) and Patrick et al. (2015) developed various training programs in which participants were informed about the nature of verbal insight problems and were then instructed to use a specific procedure to solve that specific type of insight problem.

The study presented in this paper represents a conceptual development of a previous study (Branchini et al., 2015a). In that study participants working in small groups were given implicit guidance during the search phase in order to help them to analyze the spatial properties of the problems they had been presented with in terms of contraries. Contraries acted as an implicit heuristic since participants were only “primed” to consider contraries in one experimental condition and “prompted” with a vague hint in another condition. They were not specifically told how or why doing this might help. The suggestion led to shorter periods of time needed to find the solution, increased success rates and it also modified the kind of operations performed during the solution process: there were more goal directed behaviors, more reformulations of the problem and more operations directed toward a modification of the visual structure of the problem (e.g., changing orientation and localization, and reciprocal positioning of parts of the overall structure). In Gale and Ball's study too (2012), a contrast cue (ascending vs. descending triples) acted on an implicit level. Why the two exemplars should facilitate the discovery of the rules by pointing to the salient dimension (ascending-descending) was not made explicit to participants.

The study presented in this paper aimed to provide an expanded analysis of the impact of contraries on visual-spatial problem solving by foreseeing and testing the possibility that contraries might have a beneficial effect when used as part of a conscious and explicit strategy. If one keeps in mind Öllinger et al. model of the phases characterizing problem solving (Öllinger et al., 2008; but see also Ohlsson, 1992; Knoblich et al., 1999, 2001), one could foresee contraries to be beneficial in three different stages:

1. In the initial problem representation (i.e., when a “biased” problem representation is established which makes it very difficult to access the operators that are necessary to transform the problem state into a proper solution).
2. As an intentional process during the search phase (i.e., when an appropriate solution procedure might help the problem solver not to get stuck in an impasse).
3. As an unconscious process (during the impasse phase) that enables the possibility that an unconscious change in problem representation will reach the threshold of awareness.

In this paper we do not put forward a hypothesis regarding at which specific stage the effect of the training should come into play. Prompting participants to think in terms of opposites from the very beginning of the solution search phase, might have an impact already at the level of the initial representation formed in problem solver's mind, but it might also have a later effect and support representational changes following the experiences of impasse, by suggesting the “new” starting point to be considered in the new attempt (at this level it would act as an intentional process). However, the preliminary training might also activate an arousal toward oppositional thinking operating also at an unconscious level during the impasse phases. A different experimental design from that used in the present study would be needed in order to answer the question of whether the effect concerns exclusively one of these phases or all of them. The aim of this study was to verify whether the training has an effect or whether it has not.

## The present study

In this study we explore whether *explicit* training aimed at increasing the awareness of a heuristic based on contraries has a positive effect on the reasoning processes related to visuo-spatial insight problems. A brief training program was developed which demonstrated that the systematic manipulation of the features relating to a problem (in this case transforming them into their contraries) might facilitate the search for a solution. We focused on problem solving in a group setting (with groups of three people) since a previous study which demonstrated the positive effects of providing implicit guidance to use opposites in problem solving (Branchini et al., 2015a) was conducted with groups. It is also well known from previous literature that problem solving in groups does not necessarily follow the same path as individual problem solving (for a review, see for example Laughlin et al., 2006). Although our main interest was in the group condition, we also added an individual condition in order to have a comparative indication of the effect of training in this latter case.

We tested the effects of the training in terms of success rates, the time needed to find the solution and the number of attempts made in the search phase. Each drawing done by the participants in the search phase was considered an attempt to find a solution. In order to further tap into the ways in which training influenced the thought processes of the participants, we also studied the spatial characteristics of the drawings done by the groups for one of the seven problems we had given them to solve. We randomly selected the “pigs in a pen” problem. The decision was made to analyze the drawings (as a dependent variable) rather than the discussions between the participants since drawings can be regarded as behavioral correlates of the cognitive search space and as such reveal participants' aware and unaware cognitive processes (see Fedor et al., 2015). Moreover, drawings are often the best way to share thoughts when people work together in a group.

Specifically, we aimed to explore whether the training impacted on their performance in terms of:

(1) The number of problems successfully solved.
(2) The time needed to find the correct solution.
(3) The number of attempts made.

Moreover, by means of an in-depth analysis of the drawings done by the participants working in groups while trying to solve the “pigs in a pen” problem, we aimed to gain an insight into how the training impacted on:

(4) The set of properties explored in the drawings (which we take as a indication of the search space): did participants in the training condition focus on a broader *set of properties*?
(5) Whether both poles of the dimension concerned were explored (the search space in terms of dimensions): did participants in the training condition explore one *property and its contrary* more often than those in the baseline condition?
(6) The degree of *changeability/fixedness* of the properties relating to the problem: were participants in the training condition more inclined to abandon or change a property instead of keeping it fixed?

In order to help us to interpret the findings which had emerged for the analyses of the drawings in the group condition, a comparative analysis of the dimensions manipulated by participants in the individual (baseline versus training) conditions was also conducted.

## Materials and methods

### Participants

One hundred and thirty-six participants (46 male, 90 female, *M* = 25.74 years, *SD* = 2.45 years) took part in the experiment individually (62 in the baseline condition and 74 in the training condition) during university classes on topics not related to the study. Another one hundred and twenty participants (33 male, 87 female, *M* = 21.73 years, *SD* = 2.19 years) took part in the experiment in groups of three. They were divided in forty inter-observational groups (20 groups, i.e., 60 participants, in the baseline condition and 20 groups, i.e., 60 participants, in the training condition). All of the participants gave written informed consent to participate in the study and they were undergraduate students at the University of Verona. The study was approved by the ethical committee of the Department of Human Sciences of the University of Verona (Italy) and conforms to the ethical principles of the declaration of Helsinki (World Medical Association, 2013).

### Materials/problems

Seven spatial geometrical problems were used in all conditions (see Table 1). The order of the seven problems was randomized between participants.

### Procedure

The experiment consisted of two Participation conditions (individually vs. in groups) and two Training conditions (the training condition vs. the no-training condition or baseline). In the baseline condition, participants were presented with seven spatial geometrical problems and were asked to find a solution. In the training condition, participants attended a brief training session (duration: 10 min) before being shown the problems. During the training session, one of the experimenters explained how a strategy based on the manipulation of contraries could help with spatial geometrical problems. This was done by showing how three spatial geometrical problems—i.e., the “parallelogram” problem (Wertheimer, 1945), the “nine-dot” problem (Maier, 1930) and the “altar-window” problem (Wertheimer, 1945)—could be solved by applying this strategy (to understand precisely what “changing a property into its contrary” means in relation to the three example problems we refer to, see Appendix 1 in Supplementary material). The participants were then requested to apply the strategy to seven new problems (see Table 1). They were specifically invited to identify and list all the spatial features which characterized the problem and then transform them into their contraries (the first step) before embarking on the search for a solution (the second step). Before being given the seven problems, the participants were requested to rate on a 0–10 point scale how well they had understood the training and to what extent they considered it to be useful.

In all the conditions, participants were provided with pens and sheets of paper to use for drawings or notes. They were given seven and a half minutes for each problem^1^. When they thought they had found the solution, they were instructed to raise their hands. The experimenters took note of their response time before ascertaining whether the solution was correct or not. If it was, the response time was recorded, if not, they were encouraged to keep searching. All the sessions were video-recorded.

## Results

There are two points regarding methodology which need to be noted before the discussion of the results. First, the survey carried out after the training session showed that the participants exposed to training reported that they felt they had understood it (mean rating of understanding in both conditions: *M* = 7.4, *SD* = 1.67) and that they considered it to be potentially useful (mean rating of predicted usefulness in both participation conditions: *M* = 7.33, *SD* = 1.86). This confirms that the participants had not only been exposed to training, but that they had also taken it in.

Secondly, all the statistical analyses presented in the following sections were carried out using Generalized Mixed Effect Models (GLMM). This meant it was possible to deal with the variability related to the Problems and the Subjects as random effects while considering the two experimental conditions (Participation condition and Training condition) as fixed effects. Random effects have factor levels that do not exhaust the possibilities. If one of the levels of a variable were replaced by another level, the study would be essentially unchanged (Borenstein et al., 2009). For the purposes of the hypotheses tested in our study, the problems used in the experiment were simply exemplars of a general category (i.e., visuo-spatial geometrical problems) and they were interchangeable with any other problems of the same type. They did not differ in terms of one or another feature that we were interested in studying because we expected a systematic interaction between it and the fixed effects manipulated in the study; we chose these problems as random exemplars of visuo-spatial problems of varying degrees of difficulty. According to the item response theory (Baker, 2001), every item can be described by two characteristics: item discrimination and item difficulty. These express the relationship between a latent ability (in our case insight problem solving ability) and the probability of correct responses for an item (in our case, a problem). Since our study aimed to test whether the experimental conditions (Participation and Training) affected performance, one of the minimum desirable conditions to start with was to use a set of items (problems to solve) which were characterized by varying degrees of difficulty ranging in probability from 0 to 1. As can be seen in Table 2, the frequency of correct solutions associated with each problem in effect varied across problems. It was particularly high for some problems, particularly low for others and in between for some others.

**Table 2:** **Success rate (i.e., the proportion of correct responses over the total number of responses) for the seven problems used in the study, in the Training and Participation conditions**.

**Participation condition**	**Training condition**	**Problems**	**Success rate**	**Mean success rate**
Group	Baseline	Deer	0.45	0.32
Group	Baseline	Circumference	0.2	
Group	Baseline	Five-square	0.1	
Group	Baseline	Pigs in a pen	0.5	
Group	Baseline	Eight-coin	0.15	
Group	Baseline	Square	0	
Group	Baseline	Triangle	0.85	
Group	Training	Deer	0.35	0.41
Group	Training	Circumference	0.5	
Group	Training	Five-square	0.15	
Group	Training	Pigs in a pen	0.75	
Group	Training	Eight-coin	0.25	
Group	Training	Square	0	
Group	Training	Triangle	0.9	
Individual	Baseline	Deer	0.23	0.28
Individual	Baseline	Circumference	0.24	
Individual	Baseline	Five-square	0.18	
Individual	Baseline	Pigs in a pen	0.53	
Individual	Baseline	Eight-coin	0.1	
Individual	Baseline	Square	0.02	
Individual	Baseline	Triangle	0.63	
Individual	Training	Deer	0.23	0.23
Individual	Training	Circumference	0.16	
Individual	Training	Five-square	0.07	
Individual	Training	Pigs in a pen	0.53	
Individual	Training	Eight-coin	0.05	
Individual	Training	Square	0	
Individual	Training	Triangle	0.57	

### Success rates

To begin with, we studied the effects on the success rate (i.e., the number of correct responses over the total number of responses) of the Training condition, i.e., training versus baseline, and the Participation condition, i.e., individual versus group, using a GLMM (binomial family, with Subjects and Problems as random effects).

**Figure 1:**
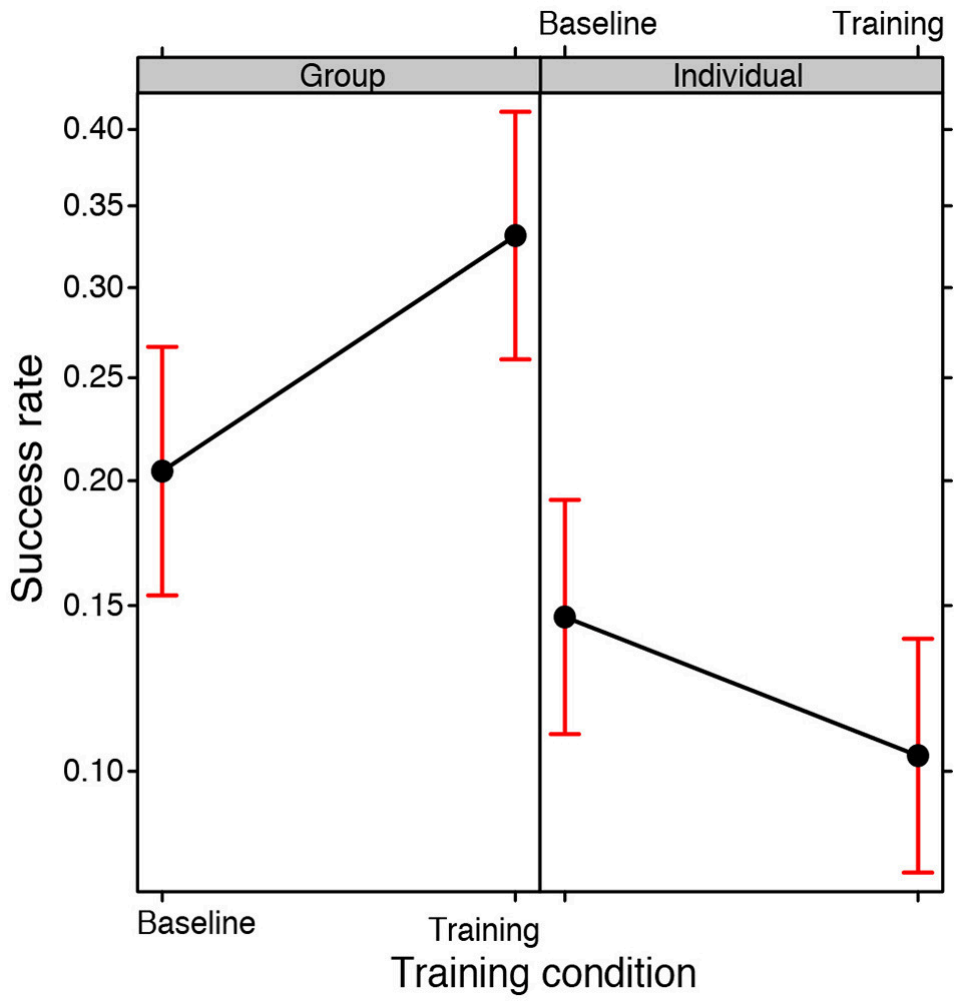
**Fixed effect plot of the interaction between the Training condition (baseline, training) and the Participation condition (group, individual) on the success rate (logit-scale)**. The bars represent 95% confidence intervals.

No significant main effect of the Training condition emerged meaning that training did not lead *per se* to better results independently of the Participation condition, i.e. in groups or individually. A main effect of the Participation condition emerged [$\chi_{\left(1,\;\text{N}\;=\;176\right)}^{2}$ = 11.6301, *p* < 0.001], suggesting that groups perform better than individuals. However, there was a significant interaction between the Participation condition and the Training condition [$\chi_{\left(1,\;\text{N}\;=\;176\right)}^{2}$ = 3.673, *p* = 0.05; see Figure 1] indicating that groups did not perform better than individuals in the baseline condition (Bonferroni *post-hoc* baseline-group vs. baseline-individual: *EST* = 0.407, *SE* = 0.389, *z ratio* = 1.044, *p* = 1.000). Therefore, being part of a group did not in itself guarantee a better success rate. Higher success rates emerged exclusively when the groups were exposed to training: their performance was significantly better than the performance of the individual participants in the training condition (*post-hoc* training-group vs. training-individual: *EST* = 1.452, *SE* = 0.384, *z ratio* = 3.778, *p* < 0.001) and also the individual participants in the baseline condition (*post-hoc* training-group vs. baseline-individual: *EST* = 1.064, *SE* = 0.387, *z ratio* = 2.744, *p* < 0.05).

### Time needed to find a solution

**Figure 2:**
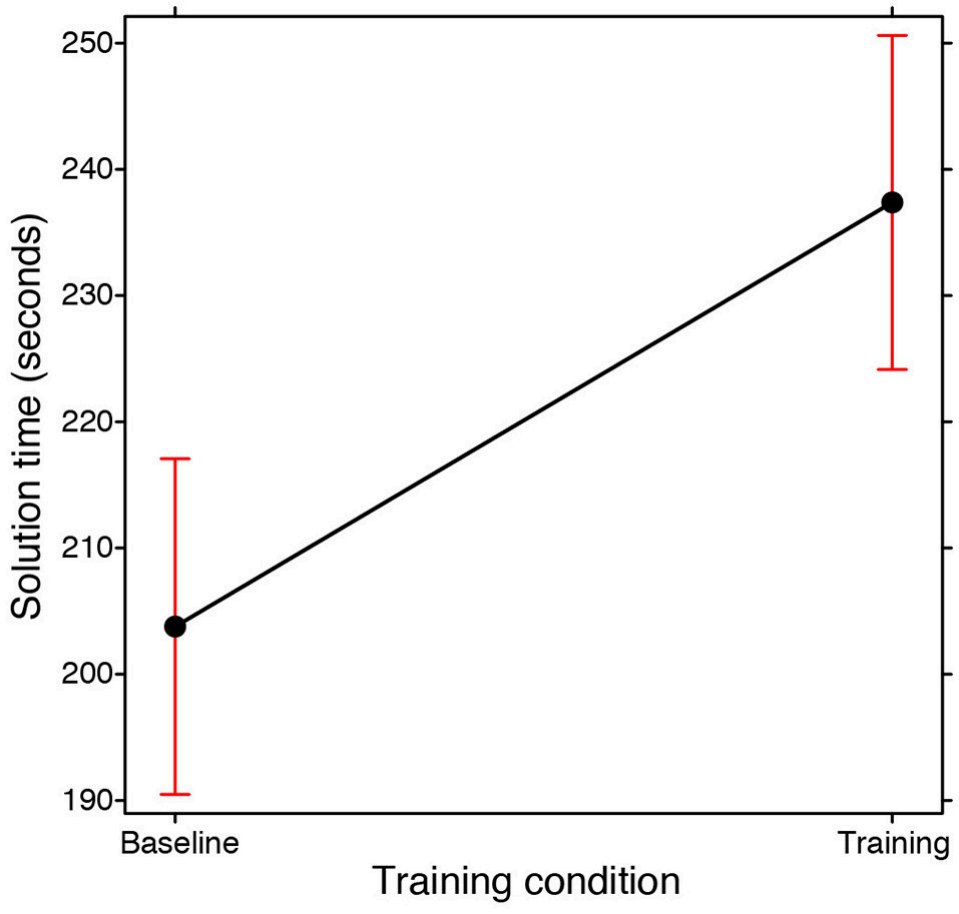
**Fixed effect plot of the main effect of the Training condition (training, baseline) on the time taken to find the solution (seconds-scale)**. The bars represent 95% confidence intervals.

A higher success rate did not necessarily mean that participants were also faster. On the contrary, a GLMM carried out on the time taken to reach the correct solution (Gaussian Family, with Training condition and Participation condition as fixed effects; Subjects and Problems as random effects) revealed that it took the participants longer to find the correct solution in the training condition than in the baseline condition [main effect of Training condition: $\chi_{\left(1,\;\text{N}\;=\;176\right)}^{2}=$ 6.144, *p* < 0.02; see Figure 2]. This was independently of whether they were working individually or in groups (i.e., no interaction between the Training and Participation conditions emerged). In the training condition they were asked to start by listing all the opposite spatial properties they could identify in the structure of the problem. As we considered this phase to already constitute part of the analysis of the problem, in the experimental design the time taken up for this analysis was included in the seven and a half minutes they had at their disposal. The longer solution times may thus be a consequence of them having spent some time on this initial phase. In other words, training is effective in terms of success rates but it is nonetheless time consuming.

### Number of attempts, i.e., the number of drawings done

**Figure 3:**
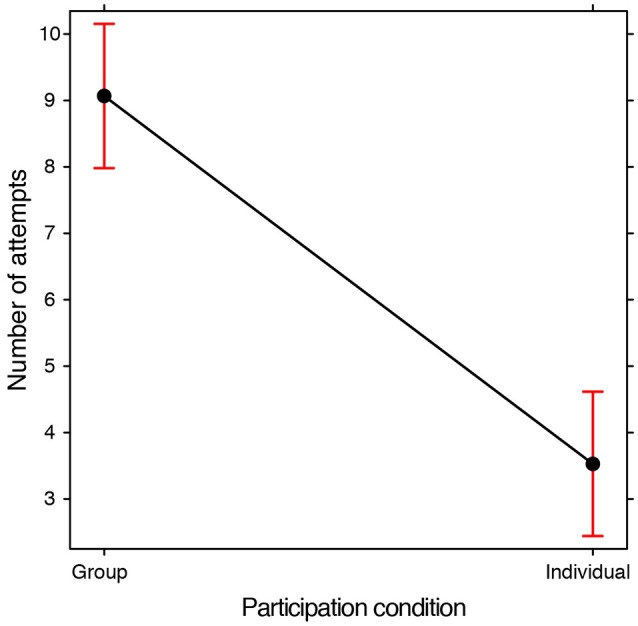
**Fixed effect plot of the main effect of the Participation condition on the average number of attempts, i.e., drawings made during the search for a solution (log-scale)**. The bars represent 95% confidence intervals.

We analyzed the number of attempts made by each group by means of a GLMM (Poisson family, with Frequency as a dependent variable, Training condition and Participation Condition as fixed effects, Subjects and Problems as random effects). There was a significant effect relating to the Participation condition [$\chi_{\left(1,\;\text{N}\;=\;176\right)}^{2}$ = 63.671, *p* < 0.0001]: in groups, participants made more attempts than when participating individually (Figure 3). There was no significant effect of the Training condition and no interaction between the two fixed effects thus indicating that training did not lead to a difference in terms of the number of attempts. The analyses which were conducted subsequently were in order to ascertain whether there were any differences in the quality rather than the quantity of the drawings.

### Behavior during the search for a solution: spatial features manipulated in the drawings done by the groups when trying to solve the “pigs in a pen” problem (in the baseline and training conditions)

As part of this study, we also examined the drawings done by the participants in their search for a solution to the “pigs in a pen” problem (randomly chosen out of the seven presented). We studied whether and how the training and baseline conditions differed in terms of the set of spatial properties explored in the drawings (Section The space relating to the problem: relevant and non-relevant properties) and whether both poles of a dimension were considered (Section The search space in terms of dimensions). We also assessed the degree of changeability/fixedness of the properties considered in each of the attempts (Section Degree of changeability/fixedness of the properties considered in each of the attempts). These analyses were meant to help explain how the training had modified the procedures followed by problem solvers in the search phase. We acknowledge the limits of an analysis conducted on only one of the seven problems. However, analyses of a single problem are not uncommon in insight-problem solving studies (e.g., Grant and Spivey, 2003; Kershaw et al., 2013; Öllinger et al., 2013, 2014). Moreover, the results which emerged from this analysis were not meant to be conclusive, our intention was merely to offer some further indications on how training might have modified the direction which the participants' search took.

Two independent judges analyzed 313 drawings and determined which spatial properties were displayed in each drawing using an *ad hoc* classification grid made up of 42 pairs of opposite spatial properties, i.e., 84 properties in total (e.g., symmetrical-asymmetrical, angular-rounded, left-right, dense-sparse; see Appendix 2 in Supplementary Material). The grid was an adaptation of a list of 37 basic dimensions characterizing direct experiences of space (Bianchi et al., 2011b) in terms of extension, shape, localization and orientation. The degree of inter-rater agreement reached by the two independent judges turned out to be very high (K di Cohen = 0.85).

#### The space relating to the problem: relevant and non-relevant properties

**Figure 4:**
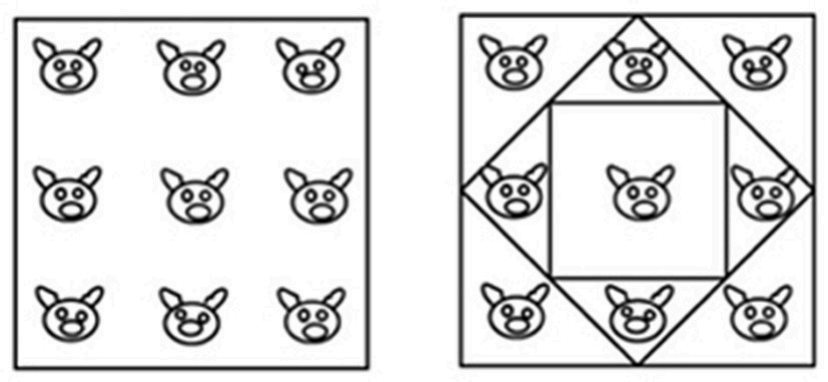
**On the left: the initial figure showing the “pigs in a pen” problem, with nine pigs enclosed within a square pen**. On the right: the solution.

The task in the “pigs in a pen” problem is to add two more square pens so as to ensure that each pig ends up in a pen of its own. The square pen shown in the initial figure is represented in its typical orientation, i.e., a square with two horizontal sides and two vertical sides (Figure 4, diagram on the left). By adding two differently oriented, progressively smaller squares inside the original pen, the solution can be found (Figure 4, diagram on the right). In terms of the classification grid used in this study, 37 of the 84 spatial properties listed are relevant (as indicated in Appendix 2 in Supplementary Material). We created a new independent variable (Relevance, with two levels: relevant, non-relevant) and analyzed the drawings done by the participants in the two Training conditions in terms of use or non-use (a dichotomous dependent variable) of the various relevant and non-relevant properties.

**Figure 5:**
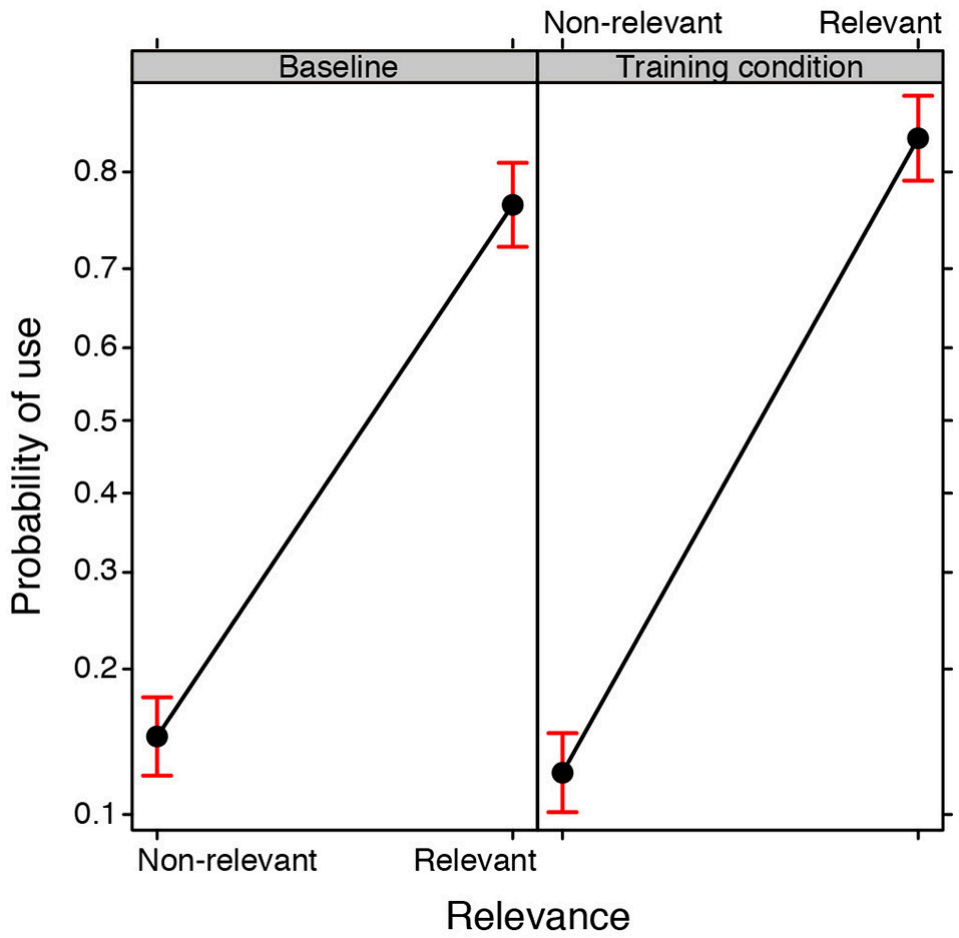
**Fixed effect plot of the interaction between the Training condition (training, baseline) and the Relevance of the properties (relevant, non-relevant) with regard to the probability of them being used (logit-scale)**. Bars represent 95% confidence intervals.

A GLMM was conducted on the Use of the 84 properties made by the groups in the baseline and training conditions (binomial family, with Training condition and Relevance as fixed effects, Group and Property as random effects). The analysis revealed a main effect of Relevance [$\chi_{\left(1,\;\text{N}\;=\;40\right)}^{2}$ = 108.173, *p* < 0.0001], i.e., relevant properties were used more frequently than non-relevant properties, but it also revealed a significant interaction between the Training condition and Relevance [$\chi_{\left(1,\;\text{N}\;=\;40\right)}^{2}\;=$ 64.725, *p* < 0.0001; see Figure 5]. *Post-hoc* tests clearly showed that relevant properties were more likely to appear in the drawings produced by the participants exposed to training as compared to the baseline condition (*EST* = 0.371, *SE* = 0.142; *z ratio* = 2.602, *p* < 0.05), whereas no significant difference was found for non-relevant properties (*EST* = 0.201, *SE* = 0.141; *z ratio* = 1.423, *p* = 0.928).

#### The search space in terms of dimensions

In the training session, it was explicitly suggested that in the search phase participants should consider not only the properties pertaining to the initial representation of the problem but also their contraries. Therefore, we expected participants exposed to training to more frequently use both of the two opposite properties in their drawings, i.e., both of the two poles forming a dimension (e.g., large and small, inside and outside) than the participants in the baseline condition.

We defined a new variable (Dimension Use) on 4 levels: Dimension Within Attempt (DWA), i.e., both properties were used within the same drawing (e.g., a straight sided square pig pen and an obliquely oriented pig pen); Dimension Between Attempts (DBA), i.e., a property was used in one drawing and the opposite property was used in another drawing (e.g., one drawing exclusively showed straight sided square pig pens and another drawing displayed an oblique pig pen); Pole (P), i.e., participants never referred to a whole dimension in any of their drawings (e.g., they drew only straight sided pens and never changed the orientation of the pen); None (N), i.e., neither of the two poles were used in any of the drawings. Since in some cases both contrary properties were relevant to the solution, in other cases only one of the two properties was relevant and in yet other cases neither of the two properties was relevant, the analyses were made taking into account the Relevance of the dimension on the three levels mentioned earlier (relevant, partially relevant, non-relevant). For each of the 42 dimensions forming the classification grid, we calculated how frequently (in proportion to the total number of drawings done) the dimension was used in one of the four modalities (DWA, DBA, N, P). We then conducted a GLMM on this data (binomial family, with Training Condition, Relevance and Dimension Use as fixed effects and Dimensions and Groups as random effects).

**Figure 6:**
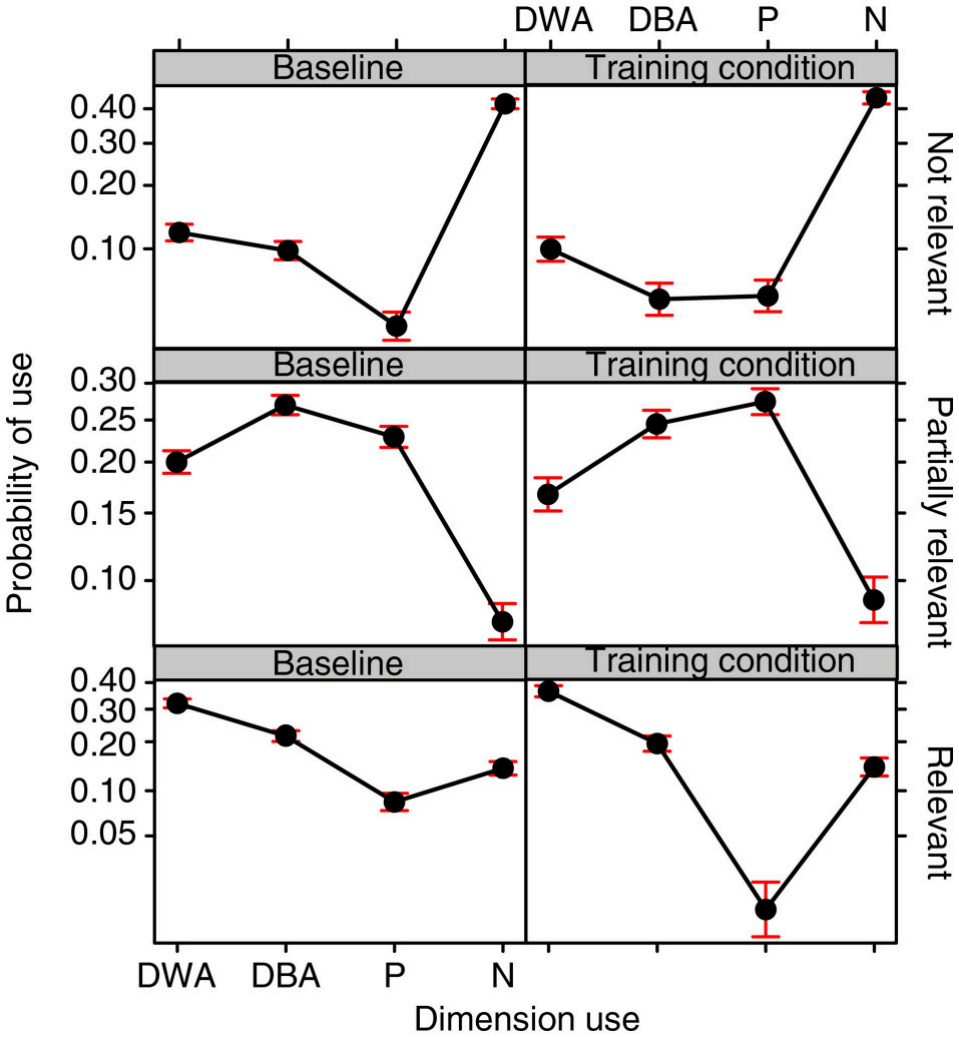
**Fixed effect plot of the interaction between the Training condition, Relevance of the properties, and Dimension use on the probability of a dimension being used (logit-scale)**. Dimension use is defined on 4 levels: Dimension displayed Within the same Attempt (DWA), Dimension displayed Between Attempts (DBA), only one Pole of a dimension used (P) and Neither of the poles used (N). Bars represent 95% confidence intervals.

The interaction between the Training condition and Dimension Use turned out to be significant [$\chi_{\left(3,\;\text{N}\;=\;40\right)}^{2}=$ 39.784, *p* < 0.0001]. Bonferroni *post-hoc* tests revealed that the drawings done in the two Training conditions did not differ either in terms of the probability of the whole dimension being used within the same drawing (DWA, *EST* = 0.077, *SE* = 0.043, *z ratio* = 1.774, *p* = 1.000) or in terms of a dimension never being used (N, *EST* = −0.083, *SE* = 0.048, *z ratio* = −1.699, *p* = 1.000). The differences we found concerned the use of only one of the two poles of a dimension (P) and the use of one dimension divided between attempts (DBA). Participants more frequently used only one pole (P) in the baseline condition as compared to the training condition (*EST* = 0.383, *SE* = 0.094, *z ratio* = 4.052, *p* = 0.001). This is in line with our prediction that training would prompt the exploration of both poles of a dimension. The use of one dimension divided between two attempts (DBA) also turned out to be more probable in the baseline as compared to the training condition (*EST* = 0.291, *SE* = 0.051, *z ratio* = 5.664, *p* < 0.0001). This is apparently in contrast with our predictions, but a significant interaction between the Training condition, Dimension Use and Relevance [$\chi_{\left(6,\;\text{N}\;=\;40\right)}^{2}=$ 104.871, *p* < 0.0001; see Figure 6] and corresponding *post-hoc* tests revealed that this held specifically for dimensions which were not relevant to the solution (*EST* = 0.621, *SE* = 0.118, *z ratio* = 5.226, *p* < 0.0001). No significant differences were found between the training and baseline conditions with regard to the relevant dimensions (*EST* = −0.124, *SE* = 0.079, *z ratio* = −1.569, *p* = 1.000) or the partially relevant dimensions (*EST* = −0.129, *SE* = 0.058, *z ratio* = −2.204, *p* = 1.000).

*Post-hoc* test also revealed that the training had reduced the use of only one pole (P) specifically for the Relevant dimensions (*EST* = 2.153, *SE* = 0.401, *z ratio* = 5.362, *p* < 0.0001). No significant differences were found between the training and baseline conditions with regard to the irrelevant dimensions (*EST* = 0.018, *SE* = 0.347, *z ratio* = 0.054, *p* = 1.000) or the partially relevant dimensions (*EST* = 0.177, *SE* = 0.324, *z ratio* = 0.547, *p* = 1.000).

#### Degree of changeability/fixedness of the properties considered in each of the attempts

**Figure 7:**
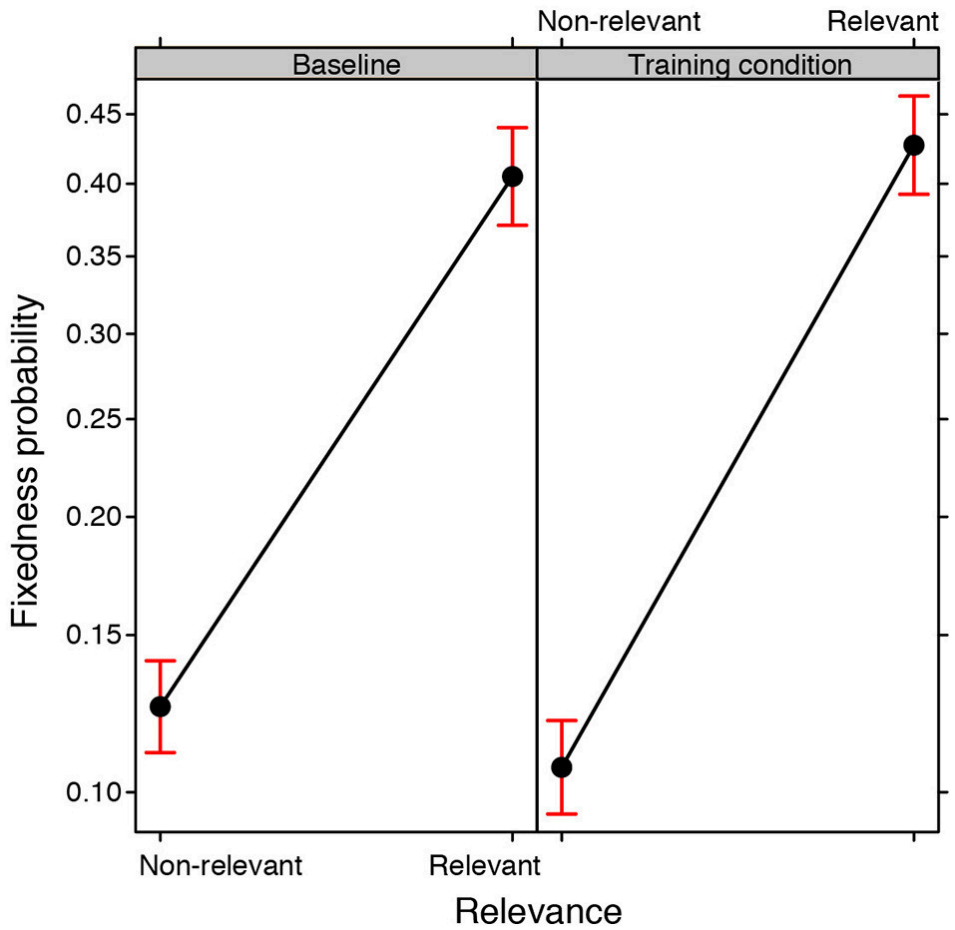
**Fixed effect plot of the interaction between the Training condition (training, baseline) and Relevance of the properties (relevant, non-relevant) on the probability of their fixedness (logit-scale)**. Bars represent 95% confidence intervals.

The changeability/fixedness of a property in the search space considered by participants was expressed in terms of the number of drawings done which displayed the property in question, as a proportion of the total number of drawings done by that group. The greater the proportional value, the greater the degree of fixedness, e.g., a value of 1 would indicate that the property was used in all of the drawings done by a particular group, representing maximum fixedness. A GLMM was conducted on the values of changeability/fixedness for each property (binomial family, with Training condition and Relevance as fixed effects, Group and Property as random effects). A significant main effect of Relevance emerged [$\chi_{\left(1,\;\text{N}\;=\;40\right)}^{2}=$ 68.268, *p* < 0.0001]: relevant properties were kept fixed more frequently across attempts than non-relevant properties. However, a significant interaction between Relevance and the Training condition also emerged [$\chi_{\left(1,\;\text{N}\;=\;40\right)}^{2}=$ 26.099, *p* < 0.0001; see Figure 7]. *Post-hoc* tests revealed that the groups exposed to training did not differ from those in the baseline condition in terms of their aptitude toward changing relevant properties (*EST* = 0.092, *SE* = 0.047, *z ratio* = 1.933, *p* = 0.319). Conversely, non-relevant properties were less fixed (in other words more changeable) across attempts in the training condition than in the baseline condition (*EST* = −0.178, *SE* = 0.058, *z ratio* = −3.081, *p* < 0.01).

### The search space in terms of dimensions manipulated in the drawings done by the participants in the individual condition when trying to solve the “pigs in a pen” problem (in the baseline and training conditions)

In order to help us explain why the effects of the training had emerged in the group condition but not in the individual condition, we explored whether the drawings made by individual participants while solving the “pigs in a pen” problem manifested similar trends to those found with the groups. In particular, we explored whether participants exposed to the training made use of only one pole of the dimension (P) less frequently than the participants in the baseline condition, or use of both of the two opposite properties more frequently in their drawings (i.e., Dimension Within Attempt, DWA, and/or Dimension Between Attempts, DBA). This might be considered a clue that they succeeded in applying the training, even though this did not lead to a higher solution rate—it is clear from literature on the subject that the effects of training are not necessarily manifested by better success rates (e.g., Patrick and Ahmed, 2014; Patrick et al., 2015).

When analysing the results of the groups, for each of the 42 dimensions forming the classification grid we calculated how frequently (in proportion to the total number of drawings done) the dimension was used in one of the four modalities (DWA, DBA, N, and P). We then conducted a GLMM on this data (binomial family, with Training Condition, Relevance and Dimension Use as fixed effects and Dimensions and Individuals as random effects). The interaction between the Training condition and Dimension Use turned out to be significant [$\chi_{\left(3,\;\text{N}\;=\;136\right)}^{2}$ = 16.1313, *p* = 0.001]. Bonferroni *post-hoc* tests revealed that the drawings done in the baseline and training conditions did not differ either in terms of the probability of the whole dimension being used within the same drawing (DWA, *EST* = −0.071, *SE* = 0.060, *z ratio* = −1.193, *p* = 1.000) or between drawings (DBA, *EST* = 0.102, *SE* = 0.074, *z ratio* = 1.380, *p* = 1.000), nor did they differ in terms of a dimension never being used (N, *EST* = −0.134, *SE* = 0.069, *z ratio* = −1.939, *p* = 1.000). The differences concerned the use of only one of the two poles of a dimension (P): similarly to the results found for the groups, individual participants more frequently used only one pole in the baseline condition as compared to the training condition (*EST* = 0.388, *SE* = 0.083, *z ratio* = 4.654, *p* < 0.0001). The significant interaction between the Training condition, Dimension Use and Relevance [$\chi_{\left(6,\;\text{N}\;=\;136\right)}^{2}$ = 48.940, *p* < 0.0001], and corresponding *post-hoc* tests, revealed that this held specifically for dimensions which were *relevant* to the solution (*EST* = 1.045, *SE* = 0.183, *z ratio* = 5.691, *p* < 0.0001). No significant differences were found between the training and baseline conditions with regard to the other categories of responses (DBA, DWA, and N). Therefore, in both the group and individual conditions, the training led to a reduction in the *partial* explorations of the solution space in terms of relevant properties (i.e., those limited to only one property, P). A second GLMM was conducted to compare the two participation conditions (with Participation condition, Training Condition, Relevance and Dimension Use as fixed effects and Dimensions and Groups as random effects). A significant interaction between Participation Condition, Training condition and Dimension Use emerged [here are the Chi square values: $\chi_{\left(3,\;\text{N}\;=\;136\right)}^{2}$ = 32.7819, *p* < 0.0001]. The use of only one pole (P) was significantly less frequent when participants exposed to the training solved the problems in groups as compared to when they did it individually (*EST* = 0.448, *SE* = 0.109, *z ratio* = 4.108, *p* < 0.005).

## Discussion and conclusions

The study presented in this paper aimed to further explore the hypothesis that reasoning in terms of contrast/contrariety/opposition might facilitate problem solving. Our results from the explicit guidance condition add to the previous literature based on implicit guidance which we mentioned in the introduction (e.g., Gale and Ball, 2012; Branchini et al., 2015a). The participants in our study were exposed to a brief training session in which it was suggested that they approach the task by systematically transforming the spatial features of a problem into their contraries. Examples were provided in order to demonstrate how this strategy might help to guide the participants' exploration of new representations throughout the solution search phase. Participants were then asked to transfer the strategy they had learned to seven other problems.

Four main findings emerged from the analyses. First, in terms of success rates (i.e., the number of problems which participants were able to find a solution to), the groups exposed to training performed better than the individuals. Exposure to training did not lead to an increase in the number of attempts made (i.e., the number of drawings). Our in-depth analysis of the characteristics of the drawings the participants had completed when trying to solve the “pigs in a pen” problem revealed that the search space which they had concentrated on did not in general expand, but they focused more on properties which were relevant to the solution, while at the same time the properties that they had examined and were non relevant to the solution were more readily disregarded in subsequent drawings. Moreover, the participants exposed to training made fewer “incomplete” explorations of the possible manipulations of the relevant properties related to the structure of the problem by limiting their explorations to only one pole. This last finding, in particular, was tested and verified in both the group and individual participation conditions.

In conclusion, our in-depth analysis of the effects of training in the case of the “pigs in a pen” problem suggests that in the group condition the training expanded the search space in a focused way, i.e., it did not lead to a disoriented multiplication of attempts and participants kept close to the properties which were relevant to the problem (on the relationship between “antonymous reasoning” and *originality* of solutions, rather than *fluency*, see also Dumas et al., 2016). We interpret this focused process (which is in line with Öllinger et al., 2013, but also Gale and Ball, 2012) as a consequence of the element of continuity that is implied in the idea of contrariety, as we pointed out in the introduction. In terms of continuity versus discontinuity in reasoning processes activated by thinking in terms of opposites, our results support discontinuity as the participants in the training condition were more likely to investigate various different paths (i.e., they less frequently limited their transformation to only one pole of the spatial dimensions they explored) each time discarding non relevant properties. They also kept more relevant properties fixed across the attempts and here too continuity is implied. Further investigations are needed in order to ascertain the extent to which these last results (which are based on an in-depth exploration of only one problem) can be generalized. What emerged is, however, in agreement with studies that suggest that the search phase of problem solving is evolutionary in nature with several search processes being launched simultaneously and their results being tested against a criterion of success which is defined by the structure of the problem. The most promising candidates are copied and modified until a solution is found or a dead-end is reached (Fernando et al., 2010; Dietrich and Haider, 2015).

The training we exposed participants to was not specific to a given problem (as in, for example, Chronicle et al., 2001, Experiment 3; Weisberg and Alba, 1981; Grant and Spivey, 2003; Kershaw and Ohlsson, 2004; Kershaw et al., 2013; Öllinger et al., 2013, 2014) but rather provided advice on how to search for a solution to a set of (spatial) problems and in this sense it resembles meta-cognitive training. However, it differs from other types of domain-specific meta-cognitive training investigated in previous literature (some of which is domain specific, e.g., Walinga et al., 2011; Patrick and Ahmed, 2014; Patrick et al., 2015) in that the participants in the present study were asked to use the “oppositional reasoning” strategy they had been told about in their exploration of the spatial domain. For both of these reasons it can be said to represent yet another method to add to the types of training whose facilitating effects on insight problem solving have been tested. It should be clear from the experimental design adopted in our study (with the control condition being no training and not another type of training) that the goal of the study was not to verify whether prompting participants to think in terms of opposites is more effective as compared to other types of training. Our goal was to collect evidence of whether explicitly showing participants how thinking in terms of contraries supports representational change leads to a better result than leaving them to work on their own. We wanted to ascertain whether this type of advice is useful and in fact the results of our study were positive.

As stated in the introduction, the study presented in this paper represents a conceptual development of a previous study in which contraries were used as an unaware, implicit strategy (Branchini et al., 2015a). A comparison between the effects of providing contraries as an implicit versus explicit guidance might tell us something more about how this heuristic impacts on the solution process. It would also be interesting to explore new ways of stimulating both implicit processing (e.g., using dynamic visual tasks) and explicit processing (e.g., using different types of training). In terms of the current state of the art situation, only a provisional comparison can be made between the improvement due to prompting participants to use contraries in implicit and explicit guidance conditions based on the findings from the present study and that carried out by Branchini et al. (2015a). There are obvious limits when one compares two experiments which do not perfectly coincide in terms of their experimental design. The problems used in Branchini et al. (2015a) were of a similar type to those used in the present study but only two were exactly the same; moreover, in the previous study the participants had no time limits whereas in the present study they were given seven and a half minutes. These differences are reflected in the percentage of correct responses in the baseline conditions in the two studies: 32% in the present study versus 67% in Branchini et al. (2015a). However, if we compare the improvement in the success rates associated with the experimental conditions in both of the studies, a similar effect emerges. Providing implicit guidance (as in Branchini et al., 2015a) led to 79% of correct solutions, which means an increment of 12% with respect to the corresponding baseline. Providing explicit guidance (as in the present study) led to 42% of correct solutions, which means an increment of 10% with respect to the corresponding baseline. The similarity between these two increments is thought-provoking. It might indicate that aware or unaware processing of contraries led to similar results or, alternatively, it might indicate that it was not the explanation given to participants about which mechanism to apply that was relevant in the training condition. The participants were asked to look for contraries before embarking in the solution process (and this is exactly the same as in the implicit guidance condition in the study done by Branchini et al., 2015a), and this, rather than the training as a whole, might have implicitly stimulated the expansion of the search space and the relaxation of the constraints relating to the mental representation of the problems.

A further question raised by the findings of the experiment presented in this paper concerns why training had positive effects specifically in the group condition. Participants worked in small groups also in the previous study where a positive effect of an implicit prompt to use opposites was found (Branchini et al., 2015a). The fact that groups work more effectively than individuals in problem solving also emerged in Augustinova's study (2008) on the benefits of *falsification cueing* in Wason's selection task, and in Laughlin et al.'s studies on letters-to-numbers problems (Laughlin et al., 2002, 2003). Attention has been devoted to how groups process information and a key factor seems to be the high degree of social sharedness of information at group level (e.g., Larson and Christensen, 1993; Wittenbaum and Stasser, 1996; Tindale and Kameda, 2000; Tindale et al., 2001; Galinsky and Kray, 2004). In order to help us to explain why, in our study, the training specifically affected success rates in the performance of the groups, but not in the performance of the individuals, we explored the dimensions manipulated in the drawings made by participants in the individual condition and matched them to those made by the groups [Section The search space in terms of dimensions manipulated in the drawings done by the participants in the individual condition when trying to solve the “pigs in a pen” problem (in the baseline and training conditions)]. We found that—similarly to what happened in groups—also in the individual condition, participants exposed to the training limited their explorations of the properties relevant to the solution to only one pole (P) less frequently than individuals in the baseline condition. Therefore, at least for the “pigs in a pen problem,” the training seems to have a similar effect in both the individual and group conditions. It was simply stronger in the latter case. Moreover, when they were in groups, the participants made more attempts than when participating individually (Section Number of attempts, i.e., the number of drawings done). These two results taken together suggest that the difference in success rates between individuals and groups might actually lay in the fact that individuals made *fewer* solving attempts than groups (although in the right direction), rather than in the fact that the attempts made to apply what they had learned were *less effective*.

Three further considerations on the effects of “thinking in opposites” in group can however be put forward. Firstly, the general hypothesis underlying our training is that referring to contraries helps people to deal with the complexity of the problem structure by showing them “what is in there,” not only in terms of actual properties but also in terms of their potential variations. The observation that every variation occurs within the framework of contraries is not only an intuition that we are indebted to Aristotle for (ed. Aristotle, 1984, Cat. 5, 4a 30-34). It is a principle that models the human direct experience of space (as pointed out by Savardi and Bianchi, 2009; Bianchi et al., 2011b) and also goes well beyond that, as testified by the pervasiveness of antonyms in every natural language (e.g., Jones, 2002; Murphy, 2003; Paradis et al., 2013). The training session in our study aimed to prompt divergent thinking and the creation of alternative representations by suggesting changes to the structure of the problem which were radical but at the same time also anchored to it. Working in groups might have facilitated this process since it has been demonstrated that the information which is more likely to be brought up in the discussion and more likely to influence decisions made is that which is shared by all group members (e.g., Wittenbaum and Stasser, 1996); the structure of the problem *is the information shared* by everyone in the group.

Secondly, in the training session participants were advised, as a first step, to identify all the spatial features characterizing the configuration of the problem. The better the descriptive analyses conducted in this initial phase were (in terms of exhaustiveness and precision), the richer the list of constraints to be relaxed in the following steps was when they transformed each feature into its contrary. We know from research into the Psychology of Perception that inter-observation in small groups of three to four members leads to more accurate descriptions of the facts under observation (see Bozzi, 1978; Bozzi and Martinuzzi, 1989; Kubovy, 2002). Therefore, in our case working in small groups might have improved the quality of the initial analysis of the structure of the problem.

Lastly, our training consisted of prompting participants to explore the structure of the problem in disconfirmatory rather than confirmatory terms. Confirmation biases are more likely to be prominent when people use their own problem solving strategies in an individual condition. On the contrary, if people in groups are asked to think in terms of opposites, not only do they do so on an individual basis, they also apply this strategy to suggestions coming from other members of the group. Moreover, it has already been shown that in argumentative discourse the ability to address *opposing positions* is crucial in order for people to coordinate their own perspective to that of other people (Kuhn and Udell, 2007) and that groups benefit more than individuals from the use of *falsification cueing* in reasoning (Augustinova et al., 2005; Augustinova, 2008). Whether these data, taken together, are general evidence that small groups provide a better context for “thinking in terms of opposites” is an intriguing question, but as yet it is still premature for conclusions to be drawn.

## Ethics statement

The study conforms to the ethical principles of the declaration of Helsinki and was approved by the ethical committee of the Department of Human Sciences of the University of Verona. Participants volunteered in the study. They signed the informed consent form approved by the ethical committee of the Department of Human Sciences of the University of Verona.

## Author contributions

EB, and EC substantially contributed to the conception of the work, the acquisition and coding of the data, the revision of the literature on the topic and the drafting of the study. IB, RB, and US substantially contributed to the conception and design of the study and the interpretation and analysis of the data. They also contributed to the drafting and revision of the work. EB, IB, RB, EC, and US approved the final version to be published and agree to be accountable for all aspects of the work in terms of the accuracy or integrity of any part of the study.

### Conflict of interest statement

The authors declare that the research was conducted in the absence of any commercial or financial relationships that could be construed as a potential conflict of interest.

## Footnotes

^1^Six to ten minutes have been used to test insight problem solving in a thinking aloud condition, for example, in studies done by Ball et al. (2015), Schooler et al. (1993) and Fleck and Weisberg (2013).
