# Supplementary material for: Can Contraries Prompt Intuition in Insight Problem Solving?
Source: Front Psychol. 2016 Dec 26;7:1962. doi: 10.3389/fpsyg.2016.01962 (PMC5183583; doi:10.3389/fpsyg.2016.01962)
Supplement: Supplementary file 1 [file DataSheet1.pdf]

Appendix 1- Training

You are taking part in an experiment which aims to study people's ability to solve visuo-spatial problems when certain suggestions are provided. We will now give you these suggestions and we advise you to use them when you are looking for a solution to the problems we will present to you.

Often the solution to visuo-spatial problems can be found by focusing on the perceptual properties of the problem and transforming some of these properties into their opposites. We will show you some examples to illustrate what we mean.

Let's look first at the "Parallelogram problem" in which there is a figure (**Figure 1**) and the task is to discover how to calculate the area of the figure and explain why this is a correct method.

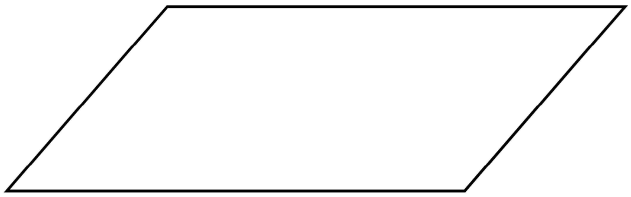

Figure 1

It's not necessary to remember the formula relating to the area of a parallelogram. You simply need to look carefully at the figure. Let's analyze it together: the figure has two long, horizontal, parallel sides and two short, oblique, parallel sides. The two long horizontal sides are not "aligned" one above the other since the figure "leans" to the right. In order to find the solution, we must find a way to "straighten" the figure out. This can be done by drawing two perpendicular, vertical lines down from the top two vertices of the parallelogram (see **Figure 2**). We can see that the triangle that now stands out on the left is exactly the same size as the triangle that seems to be missing on the right. If we move the triangle on the left so that it covers the triangle on the right, we obtain a rectangle. It is then relatively easy to calculate the area of the figure by multiplying the horizontal side by the vertical side.

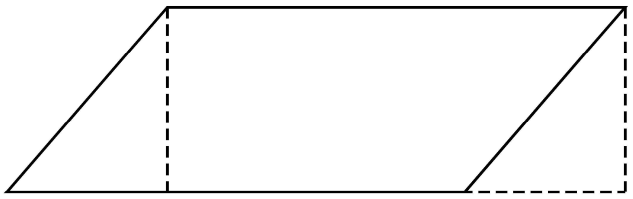

Figure 2

Operationally speaking, a strategy that we can apply is to start with the evident properties of the figure and then reason by seeing if it is possible to transform these into their contraries.

- The figure has long horizontal sides. Can I find the solution by making them vertical? (In this case no, I can't.)
- The figure has parallel sides. Can I find the solution by making them diverge or converge? (In this case, no I can't.)
- The figure leans to one side. Can I find the solution by straightening the figure? (In this case, yes I can)
- The long sides are misaligned. Can I find the solution by aligning them? (In this case, yes I can)

Note that the FIRST transformation that comes to mind may not necessarily be the one that leads to success. But by applying this strategy, it helps us to overcome a block in our reasoning process and to discover the critical transformation which will lead to the solution.

Another example is the nine dots problem (**Figure 3**). The task is to connect all the dots using four straight lines, without taking the pencil from the paper and without passing twice over the same dot.

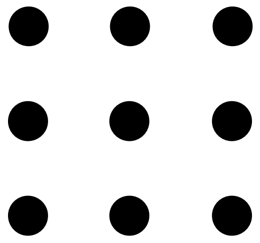

Figure 3

Look at the figure. The nine dots are lined up in three rows and three columns. Together they form a square. When we start to look for the solution to the problem, we tend to think we must join the dots either horizontally or vertically while remaining inside the square. Instead the solution requires us to do the opposite, i.e. draw lines that are oblique as well as horizontal or vertical and use lines which also go outside the square (see **Figure 4**).

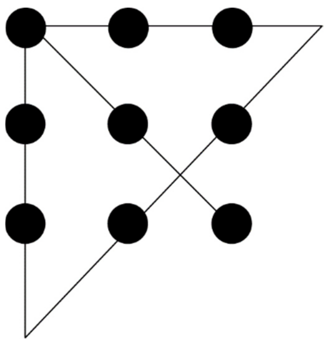

Figure 4

Again, operationally speaking, a strategy that we can apply is to start from the evident properties of the figure and then reason in terms of contraries.

- I see horizontal and vertical rows of dots. Can I find the solution by drawing oblique lines as well as horizontal and vertical lines? (In this case yes, I can)
- The dots are inside the square. Can I find the solution by drawing lines which go outside the square? (In this case, yes I can)

A third problem is the so-called Altar window problem. Workers are painting and decorating the inner walls of a church. There is a circular window a little above the altar.

As a decoration, the painters have been asked to draw two vertical lines tangentially to and of the same height as the circular window; they are then to add half circles above and below, closing in the figure.

This area between the lines and the window is to be covered with gold. For every square inch, a certain amount of gold is needed. How much gold will be needed to cover this space (given the diameter of the circle)?

The problem therefore requires us to find out how to calculate the area to be covered, as shown below (the shaded area, **Figure 5**)

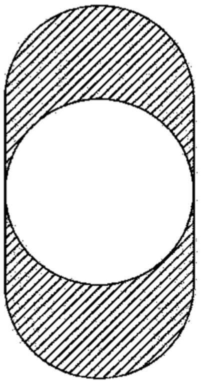

Figure 5

Let's try to apply the strategy that we have learnt.

- The figure is curvilinear. What if the solution requires us to draw straight lines?
- The circle in the middle is empty. What if the solution involves filling that empty space?
- The figure develops vertically above and below the circle. What if the solution requires us to draw horizontal lines?
- The area to be covered is uniform. What if the solution requires us to divide it into parts?

The solution is shown in **Figure 6**: if we draw two horizontal lines, tangential to the circle and the same width as the diameter of the circle, we see that a square is formed. And we also see that the semi-circles above and below the square together form a circle of exactly the same area as the circle in the center.

The area to be covered with gold thus coincides with the area of the central circle plus the four pieces outside the circle at the corners of the square. Therefore the curvilinear area to be covered with gold coincides exactly with the area of the square.

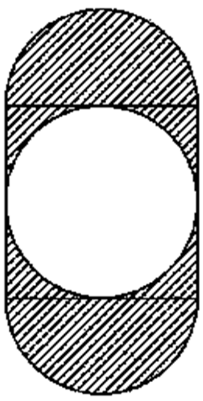

Figure 6

Appendix 2- Classification grid with pairs of visual contraries

The classification grid used in the study is based on a list of 37 basic dimensions characterizing direct spatial experience (Bianchi et al., 2011b), adapted to the "pigs in a pen" problem. This adaptation was needed to make a good fit with the contents of the drawings done by participants during their search for a solution to this specific problem. The pairs of properties (dimensions) in the classification grid do not refer to linguistically canonical antonyms, but to properties which are visually contrary. For example, the transformation of a typically oriented square into a square rotated 45° (*straight-oblique* in the classification grid) is a transformation that is frequently perceived as being contrary both by children aged 7-9 and adults (see Bianchi and Savardi, 2006, 2008c).

In this appendix, we first list the properties that make up the classification grid, then we present some examples of their application to drawings done by the participants in the study. Most of the dimensions in the classification grid concern characteristics relating to the pig pens (shape, extension, localization and orientation), others are related to characteristics regarding the pigs (number and localization), one refers to the shape of the overall configuration and one refers to the localization of both pigs and pens. The classification not only takes into account the final product (drawing) but also how it was done (e.g. the direction of the gestures made while drawing which were observed by means of the video-recordings).

Table 3. List of properties forming the classification grid.

SHAPE OF PIG PENS: unbounded–bounded; rounded–angular; incomplete–complete; curved–rectilinear; open–closed; acute–obtuse; ordered–disordered; oblong–equilateral; leaning–right-angled; stable–unstable; divergent–convergent; same shape as the original (i.e. square) –different shape as compared to the original (i.e. triangle); continuous line–broken line.

EXTENSION OF PENS: wide–narrow; large–small; whole–half.

LOCALIZATION OF PENS: right–left; inside–outside; totally outside–partially outside; totally inside–partially inside; up–down; above–below; at the top–at the bottom; intersected–non-intersected; containing–contained; centered–decentralized; attached–detached; near–far.

ORIENTATION OF PENS: standing–lying down; vertical–horizontal; oblique–straight; same orientation as the original–different orientation as compared to the original (e.g.rotated); lined up with the original frame–uneven/misaligned with respect to the original frame; parallel–perpendicular.

NUMBER OF PIGS: full–empty; dense–sparse; many (i.e. the pigs are many, as in the original pen)–few (i.e. in each of the added pens the pigs are few, namely one).

LOCALIZATION OF PIGS: widespread–localized; united–separated; still–shifted (this pair of contraries refers to whether the pigs were kept in the same position as in the initial figure or whether they had shifted).

SHAPE OF THE OVERALL CONFIGURATION: asymmetrical–symmetrical.

LOCALIZATION OF PENS AND PIGS: aligned–non-aligned.

In order to exemplify the coding system that we used, below are six of the 313 drawings produced by participants during the search for a solution and the related properties as coded by the two independent judges [see Section Behavior during the search for a solution: spatial features manipulated in the drawings done by the groups when trying to solve the "pigs in a pen" problem (in the Baseline and Training conditions)].

The first drawing represents the solution. The properties relevant to the solution appear first in the table, followed by those which are not relevant.

			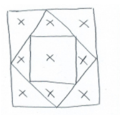	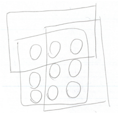	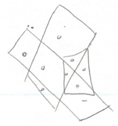	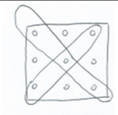	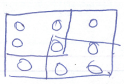	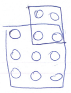
**RELEVANT PROPERTIES**	Conf.	Shape	Symmetrical				Symmetrical	
	Pens	Shape	Bounded	Bounded	Bounded	Bounded	Bounded	Bounded
			Angular	Angular	Angular	Angular	Angular	Angular
			Complete	Complete	Complete	Complete	Complete	Complete
			Closed	Closed	Closed	Closed	Closed	Closed
			Equilateral	Equilateral	Equilateral	Equilateral	Equilateral	Equilateral
			Right-angled	Right-angled	Right-angled	Right-angled	Right-angled	Right-angled
			Stable	Stable	Stable	Stable	Stable	Stable
			Unstable		Unstable	Unstable		
			Same as the original		Same as the original		Same as the original	Same as the original
			Different from the original	Different from the original	Different from the original	Different from the original	Different from the original	Different from the original
		Ext	Large	Large	Large	Large	Large	Large
			Small	Small	Small	Small	Small	Small
		Localization	Inside				Inside	
			Totally inside				Totally inside	
			Non-intersected				Non-intersected	
			Containing					
			Contained				Contained	
			Centered					
			Attached	Attached	Attached	Attached	Attached	Attached
			Near	Near	Near	Near	Near	Near
			Non-aligned	Non-aligned	Non-aligned	Non-aligned	Non-aligned	Non-aligned
		Orientation	Oblique		Oblique	Oblique		
			Straight	Straight	Straight	Straight	Straight	Straight
			Same as the original		Same as the original		Same as the original	Same as the original
			Different from the original	Different from to the original	Different from the original	Different from the original		
			Lined up				Lined up	
			Uneven/ misaligned			Uneven/misaligned		
	Pigs	Amount	Full	Full	Full	Full		Full
			Dense	Dense	Dense	Dense		Dense
			Sparse		Sparse	Sparse		
			Many	Many	Many	Many		Many
			Few	Few	Few	Few		Few
		Localization	Widespread	Widespread	Widespread	Widespread		
			Localized	Localized	Localized	Localized		
			Separated	Separated	Separated	Separated		
			Still	Still	Still	Still		
			Aligned	Aligned		Aligned		

			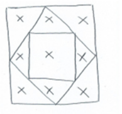	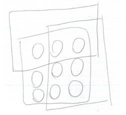	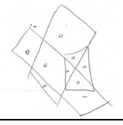	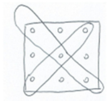	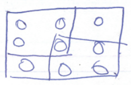	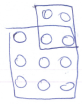
NON-RELEVANT PROPERTIES	Conf.	Shape		Asymmetrical	Asymmetrical			Asymmetrical
	Pens	Shape		Oblong	Oblong	Oblong		Oblong
					Leaning	Leaning		
						Rounded		
		Ext.		Wide	Whole			Whole
				Narrow	Half			Half
		Localization		Right	Right	Right	Right	Right
				Partially outside	Partially outside	Partially outside	Up	Partially outside
				Partially inside	Partially inside	Partially inside	Intersected	Partially inside
				Up	Up	Up	Decentralized	Up
				Intersected	Intersected	Intersected	Down	Intersected
				Decentralized	Decentralized	Down	Left	Decentralized
					Down	Left		
		Orientation		Standing				Lying down
				Lying down				Horizontal
				Vertical				
				Horizontal				
				Perpendicular				
	Pigs	Localization		United	Shifted		United	United
					Non-aligned			Shifted
